# Exploiting Protected Maleimides to Modify Oligonucleotides, Peptides and Peptide Nucleic Acids

**DOI:** 10.3390/molecules20046389

**Published:** 2015-04-10

**Authors:** Clément Paris, Omar Brun, Enrique Pedroso, Anna Grandas

**Affiliations:** Departament de Química Orgànica i IBUB, Facultat de Química, Universitat de Barcelona, Martí i Franquès 1-11, 08028 Barcelona, Spain; E-Mails: clementparis@ub.edu (C.P.); o.brun@ub.edu (O.B.); epedroso@ub.edu (E.P.)

**Keywords:** protected maleimides, oligonucleotides, peptides, conjugates, cyclization

## Abstract

This manuscript reviews the possibilities offered by 2,5-dimethylfuran-protected maleimides. Suitably derivatized building blocks incorporating the *exo* Diels-Alder cycloadduct can be introduced at any position of oligonucleotides, peptide nucleic acids, peptides and peptoids, making use of standard solid-phase procedures. Maleimide deprotection takes place upon heating, which can be followed by either Michael-type or Diels-Alder click conjugation reactions. However, the one-pot procedure in which maleimide deprotection and conjugation are simultaneously carried out provides the target conjugate more quickly and, more importantly, in better yield. This procedure is compatible with conjugates involving oligonucleotides, peptides and peptide nucleic acids. A variety of cyclic peptides and oligonucleotides can be obtained from peptide and oligonucleotide precursors incorporating protected maleimides and thiols.

## 1. Introduction. Maleimide-Involving Click Conjugation Reactions with Oligonucleotides and Polyamides

**Scheme 1:**
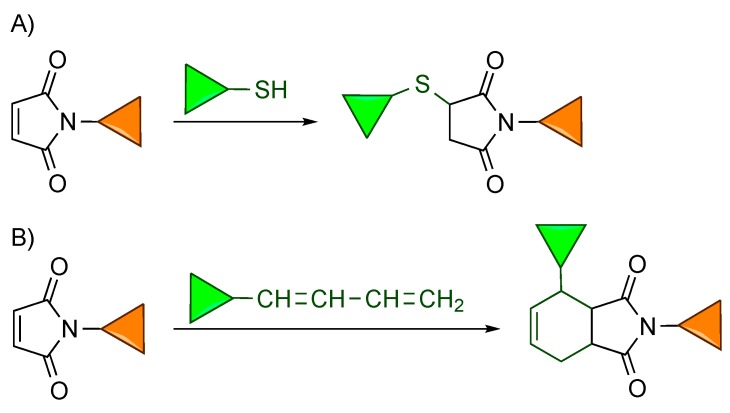
Reaction of maleimides with thiols (**A**) or conjugated dienes (**B**) provides conjugates. The two differently colored triangles represent either two different biomolecules or one biomolecule and another compound, such as a fluorophore, a spin-label or a metal complex.

The Michael-type addition of thiols to electron deficient carbon-carbon double bonds is one of the oldest click reactions [1]. It takes place quickly and in high yield, does not generate byproducts, and can be carried out in water. When applied to bioconjugations, maleimides are the electrophiles most commonly used (Scheme 1A). The maleimide-thiol conjugation chemistry has found application in the derivatization of all kinds of biomolecules, and in particular in the synthesis of the two antibody-drug conjugates approved by the FDA and presently in clinical use [2].

More recently, conjugates have also been prepared by exploiting the Diels-Alder reaction between maleimides and conjugated dienes (Scheme 1B). This cycloaddition is another modular reaction that can link two different compounds in water without producing side products; although, it does not proceed as rapidly as the thiol-maleimide reaction.

With the exception of thiols that may be present in peptides and proteins, none of the functional groups required for the Michael-type and Diels-Alder conjugation reactions exist in biomolecules. This is both an advantage and a disadvantage. An advantage because it ensures regioselectivity, in other words, it allows the click conjugation reaction to provide a chemically defined conjugate instead of a mixture of products. The disadvantage is that the conjugate components must be modified to introduce the additional functional group required for conjugation.

In regards to derivatization with maleimides, they are most often attached to biomolecules making use of bifunctional compounds that incorporate the maleimide moiety and a carboxyl group (or an active ester), of which the latter is expected to form an amide bond with amino group(s) on the biomolecule (peptide, protein). Therefore, molecules lacking or having poorly reactive amines require an additional pre-derivatization step. This is the case of oligonucleotides.

**Scheme 2:**
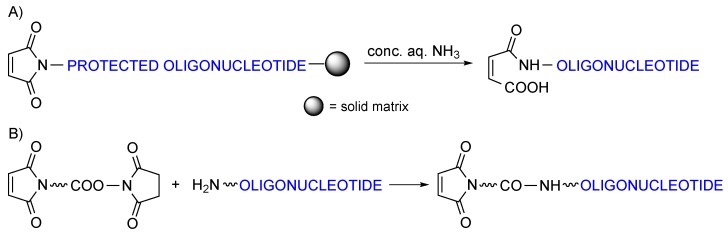
(**A**) Imide hydrolysis taking place upon treatment with ammonia of solid-phase assembled maleimido-oligonucleotides. (**B**) Most common methodology used for the attachment of maleimides to oligonucleotides in solution.

Oligonucleotides are normally prepared by solid-phase synthesis, and can be appended with alkylamines while still anchored to the solid support. Yet, the bifunctional maleimide-acid cannot be appended to the chain at this stage because maleimides do not withstand the treatment with concentrated aqueous ammonia that removes standard protecting groups and cleaves the oligonucleotide-resin linkage, even at room temperature. In addition to Michael-type addition of ammonia to the activated double bond, the imide can undergo base-promoted hydrolysis (Scheme 2A).

Derivatization of oligonucleotides with maleimides can be and has been done in solution by reacting a maleimide-acid with an amino-derivatized, fully deprotected oligonucleotide (Scheme 2B) [3,4,5,6]. This reaction is rather regioselective because the exocyclic amines on the nucleobases are poor nucleophiles, but reproducibly good yields are difficult to achieve.

**Scheme 3:**
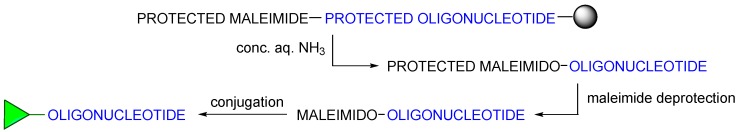
General scheme for the solid-phase synthesis of maleimido-oligonucleotides and conjugates.

We reasoned that a simple solution to this problem might be protecting the maleimide moiety (Scheme 3), so that the ammonia treatment provided the oligonucleotide chain derivatized with the protected maleimide. The protecting group obviously had to be removable under conditions not degrading the oligonucleotide chain.

## 2. Development of a Maleimide Protection Strategy Allowing Maleimido-Oligonucleotides to Be On-Resin Assembled

### 2.1. Identification of a Maleimide Protecting Group and Proof of Principle Experiments

**Scheme 4:**
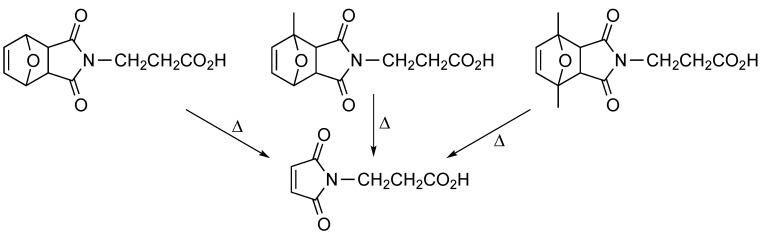
Derivatives of 3-maleimidopropanoic acid synthesized and tested to assess the extent of maleimide deprotection (retro-Diels-Alder reaction) upon heating under the same conditions.

Maleimides can be protected by reaction with dienes, which provides the corresponding cycloadduct [7,8]. Since the cycloadducts that can be reversed more easily are those involving furans, we decided to evaluate three different furans. To assess their suitability as maleimide protecting groups, furan, 2-methylfuran and 2,5-dimethylfuran were reacted with maleimidopropanoic acid, and the corresponding cycloadducts (Scheme 4) were heated to recover the unprotected maleimide-containing acid. In agreement with described results [9], the 2,5-dimethyl cycloadduct allowed for the reverse Diels-Alder reaction under milder conditions. As a result, 2,5-dimethylfuran-protected 3-maleimidopropanoic acid was used in the first proof of principle assays [10].

**Scheme 5:**
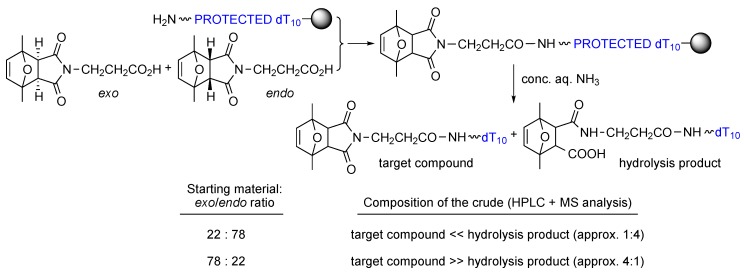
First experiments carried out with batches of 2,5-dimethylfuran-protected 3-maleimidopropanoic acid containing different *exo*/*endo* ratios showed a very good correlation between target compound and percentage of *exo* adduct in the starting material.

2,5-Dimethylfuran-protected 3-maleimidopropanoic acid was prepared carrying out the reaction between 2,5-dimethylfuran and 3-maleimidopropanoic acid either in CH_2_Cl_2_ overnight at room temperature, or in acetonitrile for 6 h at 60 °C. The two batches were used to couple the maleimide-protected acid to amino-derivatized dT_10_-resin (Scheme 5). To our initial surprise, after ammonia deprotection at room temperature, the HPLC traces of the two crudes showed the same main peaks (and products, as assessed by MALDI-TOF MS) but in different ratios (see Scheme 5). Careful inspection allowed a correlation between the ratios of *exo* adduct and target compound, which indicated that the *endo* adduct was not stable to the ammonia deprotection conditions and thus not appropriate for maleimide protection (even though it undergoes the retro-Diels-Alder reaction more easily) [11].

**Scheme 6:**
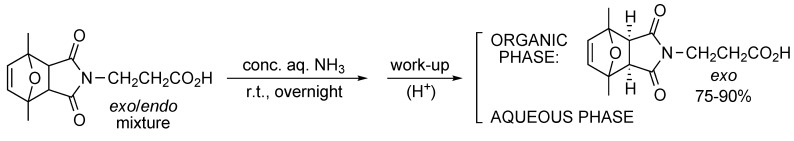
The two 2,5-dimethylfuran-protected 3-maleimidopropanoic acid isomers can be easily separated by taking advantage of the stability of the *exo* cycloadduct in aqueous basic conditions.

TLC Analysis clearly indicated that separation of the *exo* and *endo* isomers of 2,5-dimethylfuran-protected 3-maleimidopropanoic acid by column chromatography would be neither easy nor high yielding. However, we soon realized that the fact that one cycloadduct was not stable to ammonia could be advantageously exploited to separate the two cycloadducts (Scheme 6). Thus, the batch containing the *exo*-rich mixture of cycloadducts was treated with concentrated aqueous ammonia overnight at room temperature, and this was followed by evaporation of ammonia under reduced pressure, addition of trifluoroacetic acid (or aqueous HCl, see reference [10]) and extraction of the resulting aqueous solution with dichloromethane. Dichloromethane removal under reduced pressure usually provided pure *exo* adduct, as assessed by ^1^H-NMR. In case a mixture still containing *endo* adduct is obtained (generally less than 5%–10%), in particular when carrying out the separation process at the gram scale, we repeat the process (once, or even twice, if required) until complete purification is achieved. The desired product is always recovered in very high yield (Scheme 6).

We are only aware of one report in which the *endo* cycloadduct is said to be hydrolyzed in basic conditions more quickly than its *exo* counterpart [12], and to our knowledge no explanation has been ever provided for this behavior.

**Scheme 7:**
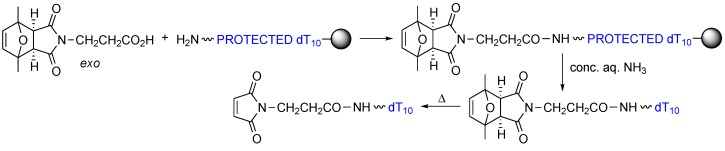
Proof of principle experiments showing that protected-maleimide-dT_10_ can be obtained with no side products from the *exo* isomer of 2,5-dimethylfuran-protected 3-maleimidopropanoic acid, and the maleimide subsequently deprotected by carrying out a reverse Diels-Alder reaction.

Pure *exo* 2,5-dimethylfuran-protected 3-maleimidopropanoic acid was then coupled to amino-derivatized dT_10_-resin (Scheme 7). HPLC analysis of the crude after ammonia treatment at room temperature showed that the target (protected maleimide)-dT_10_ had been obtained, with no product resulting from hydrolysis of the succinimide.

To deprotect the maleimide, a 1:1 (v/v) MeOH/H_2_O solution of (protected maleimido)-dT_10_ was heated in a MW oven for 60 min at 90 °C. Deprotection was not quantitative, but the crude was very clean. In subsequent experiments the reaction time has always been prolonged to 90 min (see Scheme 9), with quantitative or nearly quantitative maleimide deprotection yields (Section 2.2). It was also found that oligonucleotide concentration is an important parameter, and that the homogeneity of the crude decreases if this concentration is above 25–50 μM.

### 2.2. Experiments Broadening the Scope of Applications of the Method in the Field of Oligonucleotide Conjugates

After this successful preliminary result, the following issues remained to be addressed: (i) synthesizing a (protected maleimido)-phosphoramidite enabling to attach the protected maleimide moiety to the 5' end of the resin-linked oligonucleotide, under the same conditions as nucleoside building blocks and with no need for additional derivatization (that is without requiring incorporation of an amine group); (ii) preparing derivatives allowing for incorporation of the protected maleimide either at the 3' end or at internal positions; (iii) confirming the compatibility of MW-promoted maleimide deprotection conditions with any oligonucleotide sequence, and identifying deprotection conditions not requiring a MW oven (which is a fairly common equipment in chemistry but maybe not in chemical biology laboratories); (iv) verifying that a fully reactive maleimide is obtained after the deprotection step, yielding the target conjugate; and (v) assessing the stability of (protected maleimido)- and maleimido-oligonucleotides.

**Scheme 8:**
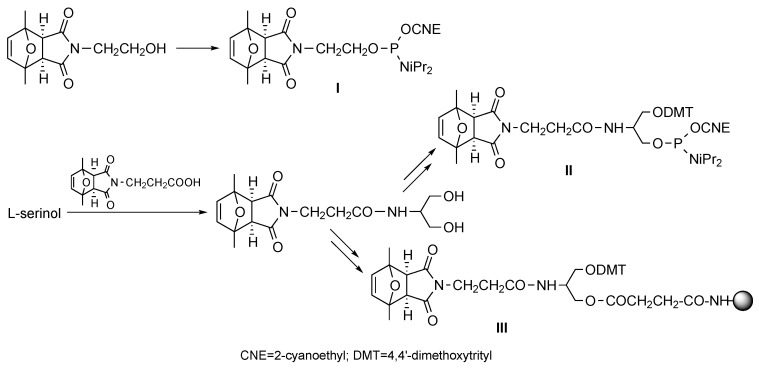
Structures of the phosphoramidite (**I** and **II**) and solid support (**III**) building blocks (and of their precursors) from which oligonucleotides derivatized with protected maleimides at the 5' end, internal positions, or the 3' end can be obtained, respectively.

The phosphoramidite enabling 5' derivatization with a protected maleimide was prepared from *N*-(2-hydroxyethyl)maleimide, which was reacted with 2,5-dimethylfuran, treated with ammonia as explained above to obtain the pure *exo* adduct, and phosphitylated (compound **I**, Scheme 8) [10]. This phosphoramidite derivative is now commercially available from Glen Research Co.

To allow for incorporation of protected maleimides at oligonucleotide positions different from the 5' end, serinol derivatives containing a protected maleimide on the amine, a DMT group on one hydroxyl, and either a phosphoramidite or a succinyl group on the other (**II** and **III**, respectively, Scheme 8) were obtained and successfully used in oligonucleotide synthesis [13].

**Scheme 9:**
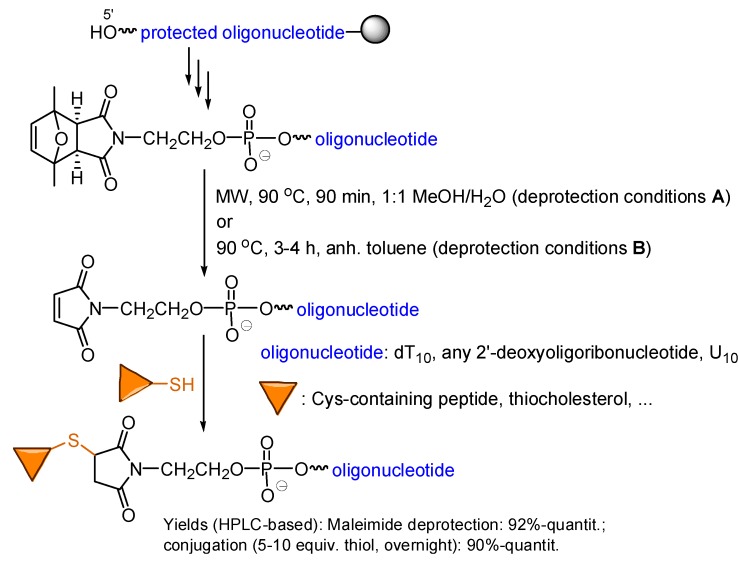
Two alternatives allow the maleimide moiety to be deprotected, furnishing maleimido-oligonucleotides that efficiently react with thiols and provide the target conjugates.

Phosphoramidite **I** was first coupled to dT_10_-resin, to yield the corresponding (protected maleimido)-oligonucleotide as a single peak after ammonia deprotection. Heating in a MW oven (90 min reaction time, see Scheme 9: deprotection conditions **A**) furnished the target maleimido-dT_10_ in very good yield (>95%).

dT_10_ is a clean crude-yielding sequence easy to work with in first, proof of principle experiments, but not necessarily providing generalizable results. For this purpose, other sequences (10-, 18- and 21-mer 2'-deoxyoligoribonucleotides with all the nucleobases, and U_10_) were assembled, derivatized with the protected maleimide using amidite **I**, and the oligonucleotide deprotected and purified. In all cases (protected maleimido)-oligonucleotides were satisfactorily obtained and deprotected.

In order to apply our method in those laboratories where a MW oven is not available, different deprotection conditions were tested, which resulted in the conclusion that heating a suspension of (protected maleimido)-oligonucleotide in strictly anhydrous toluene for 3–4 h at 90 °C also furnishes the target maleimido-oligonucleotide (deprotection conditions **B**, Scheme 9). We have not found significant differences in the crudes when using either of the two deprotection alternatives. Again, the amount of oligonucleotide in the suspension was found to be important, and best crudes were obtained when the molar amount of oligonucleotide and the volume of toluene employed would furnish a 25–50 μM concentration were the oligonucleotide soluble in toluene. We have no explanation to the fact that maleimide moieties can be safely deprotected in a MW oven when in a 1:1 MeOH/H_2_O solution, whilst heating the (protected maleimido)-oligonucleotide in slightly humid toluene promotes hydrolysis of the maleimide to an unacceptably high degree.

All the maleimido-oligonucleotides reacted with thiol-containing compounds to provide the target conjugates (Scheme 9), thus confirming that the maleimide deprotection conditions do not harm the maleimido-oligonucleotide and furnish fully reactive maleimides [10].

Very recently [14], it has been reported that (protected maleimido)-oligonucleotides assembled on an alkyl-chain-soluble support can successfully be deprotected by heating in toluene (90 min, 90 °C), and after solvent removal conjugated with a variety of thiol-containing compounds. Therefore, this methodology, routinely used in our laboratory, has also proved successful for others.

At this point, some comments have to be made in relation to stability issues. Treatments with concentrated aqueous ammonia, 0.05 M K_2_CO_3_ in MeOH and AMA (1:1 concentrated aqueous ammonia/aqueous methylamine mixture) can be used to remove the oligonucleotide permanent protecting groups provided that the reaction is carried out at room temperature. Therefore, (protected maleimido)-(protected oligonucleotide)-resins have to be assembled using phosphoramidite derivatives in which the nucleobases are protected with groups that do not require heating for deprotection, such as A^Pac^ (Pac = phenoxyacetyl), C^Ac^, and G^iPrPac^ or G^dmf^ (iPrPac = isopropylphenoxyacetyl; dmf = dimethylformamidine). (Protected maleimido)-oligonucleotides can be purified and lyophilized and safely kept in the freezer for months. They can be kept in aqueous solutions for about one week, with no great difference between standing at 15–20 °C or in a refrigerator. Maleimido-oligonucleotides are not very stable (see below), so it is highly recommended that maleimide deprotection be immediately followed by conjugation.

**Scheme 10:**
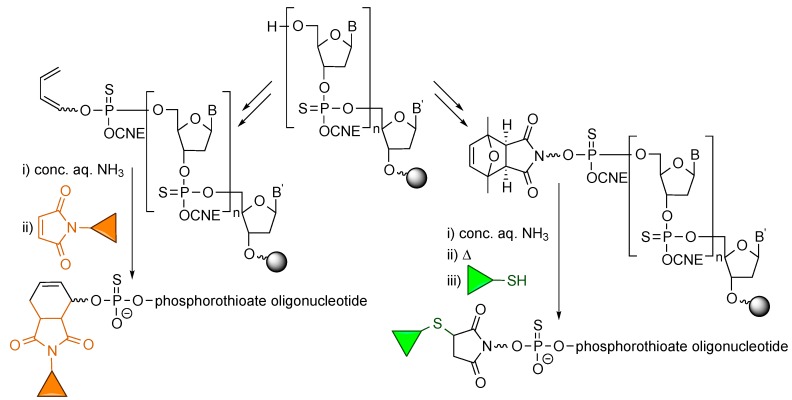
Both diene- and maleimido-derivatized phosphorothioate oligonucleotides can be reacted with maleimides and thiols, respectively, and provide the target conjugates with no undesired reaction between the thiophosphate diesters and the maleimide.

Replacement of phosphates by phosphorothioates is a modification that has been extensively used to prevent the degradation of potential oligonucleotide drugs by nucleases, in particular 3'-exonucleases. One question that troubled us was whether phosphorothioates would react with maleimides and behave as thiol competitors in the Michael-type conjugation reactions. Different experiments were carried out to address this issue, of which the most important are summarized in Scheme 10 [15]. The conclusion was that, in contrast with thiophosphate monoesters, which do react with maleimides, thiophosphate diesters do not react with maleimides. On the one hand, the Diels-Alder cycloaddition between diene-derivatized phosphorothioate oligonucleotides and maleimide-containing compounds afforded the target conjugates with no evidence of multiple incorporation of the maleimide-appending moieties onto the oligonucleotide as a result of phosphorothioate-maleimide Michael-type reactions. On the other, more challenging experiments with (protected maleimido)-phosphorothioate-modified oligonucleotides, where maleimide deprotection was followed by conjugation with a thiol, confirmed that even under the heating conditions in which the maleimide is deprotected through a retro-Diels-Alder reaction, there is no detectable maleimide-phosphorothioate reaction.

## 3. Further Applications of Protected Maleimides

### 3.1. Derivatization of Polyamides. Simultaneous Maleimide Deprotection and Conjugation Allows for Double Conjugation of Polyamides as Well as Synthesis of Oligonucleotide Conjugates

The covalent attachment of different moieties to a biomolecule may serve different goals, such as facilitating cell uptake, monitoring the fate of the biomolecule within cells, or allowing biomolecules interacting with a given target to be detected and/or captured and identified. The attachment of two moieties allows two of these aims to be simultaneously achieved.

From the synthetic point of view doubly derivatized biomolecules can be prepared in different ways, either assembling the conjugate in a stepwise manner employing building blocks that carry the desired moieties, or introducing new functional groups into the biomolecule to perform two subsequent conjugation reactions in solution, or combining both. With the purpose of carrying out two subsequent conjugation reactions in solution, we considered the possibility of exploiting the versatility of maleimide protection to attach maleimides and protected maleimides to a biomolecule (or analog). In order to broaden the scope of possible applications of protected maleimides, we decided to first explore their use in the derivatization of polyamides, in particular peptides, peptide nucleic acids (PNAs, oligonucleotide analogs made up of amide-linked *N*-(2-aminoethyl)glycine units to which nucleobases are appended) and peptoids (peptide analogs in which natural amino acids are replaced by *N*-alkylglycines).

All of these polyamides are commonly assembled by solid-phase synthesis. In the three cases the final step that removes permanent protecting groups and cleaves the polyamide-resin linkage is an acid treatment with a mixture typically containing at least 90% trifluoroacetic acid in addition to scavengers, which does not harm maleimides. However, standard peptide and PNA building blocks carry a base-labile Fmoc (9-fluorenylmethoxycarbonyl) temporary protecting group, and the simplest method to elongate peptoid chains on an insoluble matrix consists in the incorporation of chloro- or bromoacetyl units, followed by reaction with a large amount of suitably derivatized amines. Both the piperidine treatment that removes Fmoc groups and the reaction in which α-haloacyl groups are transformed into secondary amines damage maleimides. Hence, free maleimides can only be placed at the *N*-terminal position.

**Scheme 11:**
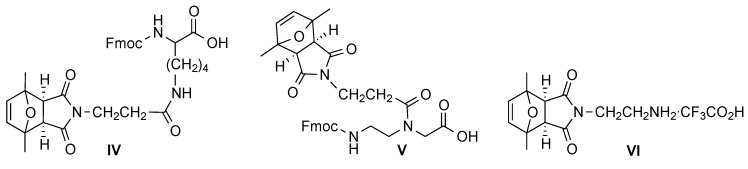
Structures of the monomers that allow protected maleimides to be introduced into peptide chains (**IV**), PNAs (peptide nucleic acids) (**V**), and peptoids (**VI**).

We hypothesized that maleimides protected with 2,5-dimethylfuran would survive both of these basic treatments, and decided to prepare monomers (**IV**, **V** and **VI**) allowing for the introduction of protected maleimides into the three types of polyamides (Scheme 11) and use them for their derivatization [16].

An important issue was assessing that all (protected maleimide)-containing polyamides could be deprotected using the previously developed procedures, and eventually identifying best maleimide deprotection conditions. MW irradiation of a 1:1 MeOH/H_2_O solution was found to be the best alternative to obtain maleimido-PNAs, whereas heating in toluene is recommended for peptides and peptoids. This reaction is troublesome in the case of peptides, since yields are low (30%–50%) and prolonging the reaction time results in crudes of increasing complexity. Yet, in all cases, the free maleimides that resulted from the retro-Diels-Alder reaction were functional and effectively yielded the target conjugates.

With the aim of increasing the deprotection yield of (protected maleimido)-peptides, we examined the possibility of simultaneously carrying out the retro-Diels-Alder reaction and the conjugation. When a (protected maleimido)-peptide was heated in the presence of a thiol, we attained a global deprotection+conjugation yield higher (80%–95%) than those of the two separated reactions (overall 25%–35%). At this point it remained to be seen whether the retro-Diels-Alder reaction that removes the maleimide protecting group and the Diels-Alder cycloaddition that allows unprotected maleimides to react with 1,3-dienes to afford conjugates could be performed simultaneously. We found that this is indeed the case, that these two reactions can be conducted simultaneously because the diene-maleimide cycloadduct is more stable than the 2,5-dimethylfuran-maleimide cycloadduct.

Subsequent experiments have confirmed that protected maleimides appending from peptides are difficult to deprotect, and that simultaneous deprotection (by heating in a MW oven) and conjugation is the best alternative to synthesize the target conjugate. Formation of the conjugate consumes the free maleimide, drives the retro-Diels-Alder equilibrium toward the deprotected maleimide, and prevents the maleimide from being degraded.

**Scheme 12:**
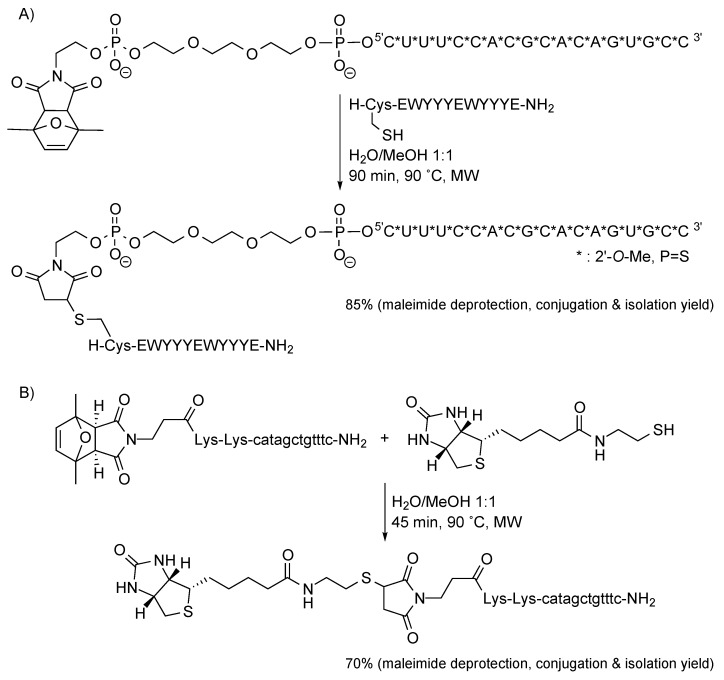
Oligonucleotide (**A**) and PNA (**B**) conjugates prepared by simultaneously carrying out the deprotection of the maleimide and the Michael-type reaction with a thiol.

Simultaneous maleimide deprotection and conjugation can also successfully furnish conjugates of oligonucleotides and PNAs, as shown in Scheme 12. In the first conjugate, a 12-mer peptide (H-CEWYYYEWYYYE-NH_2_) is attached to an 18-mer 2'-*O*-methyl, phosphorothioate oligoribonucleotide (^5'^CUUUCCACGCACAGUGCC^3'^, with a triethyleneglycol spacer between the oligonucleotide and the protected maleimide). In the second conjugate, a thiol derivative of biotin is linked to a 12-mer PNA (H-KK-catagctgtttc-NH_2_, with two lysines between the *N*-terminus and the protected maleimide to ensure solubility of the PNA chain). In both cases, after the reaction in which the oligomer-resin bond was cleaved and the corresponding oligomers were devoid of permanent protecting groups, maleimide deprotection and conjugation were carried out simultaneously, and satisfactorily afforded the target conjugates.

**Scheme 13:**
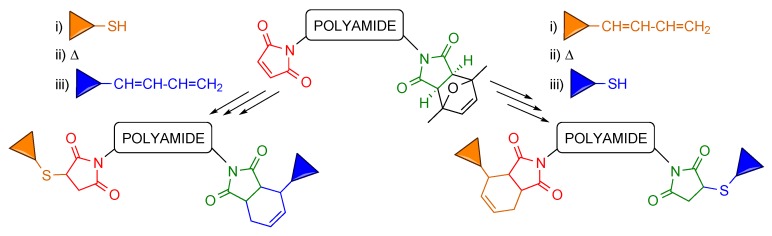
Double conjugation on polyamides (peptides, PNAs) incorporating a maleimide and a protected maleimide. The free maleimide (shown in red) undergoes a first conjugation reaction (Michael-type or Diels-Alder, with a suitably derivatized compound here represented by the brown triangle), which is followed by maleimide deprotection and a second conjugation with the compound represented by the blue triangle. Maleimide deprotection and the second conjugation can be carried out simultaneously (see above).

As shown in Scheme 13, peptides and PNAs decorated with two different moieties were obtained from double conjugations in which a first click reaction (either Diels-Alder or Michael-type) with a free maleimide was followed by maleimide deprotection and a second conjugation reaction, or in which maleimide deprotection and the second conjugation were simultaneously carried out. Cysteine-containing peptides, thiol-derivatized biotin, and diene-oligonucleotides (both with the natural phosphates and with thiophosphates) were used as decorating molecules.

Hence, the deprotection and conjugation strategy is a suitable alternative to prepare conjugates of polyamides (peptides, PNAs, peptoids) and oligonucleotides, as well as to cyclize peptides and oligonucleotides (see below).

### 3.2. Use of the Maleimide-Thiol Reaction for Cyclization

Cyclic peptides and cyclic oligonucleotides are interesting molecules because they lack free ends and their stability in biological media (in particular to exopeptidases and exonucleases, respectively) is higher than that of the linear counterparts, and because cyclization results in structures with a higher degree of spatial organization. To our knowledge, when we started this work the thiol-maleimide reaction had not been used for the cyclization of oligonucleotides, and only in one occasion to cyclize a peptide [17].

#### 3.2.1. Cyclic Oligonucleotides

**Scheme 14:**
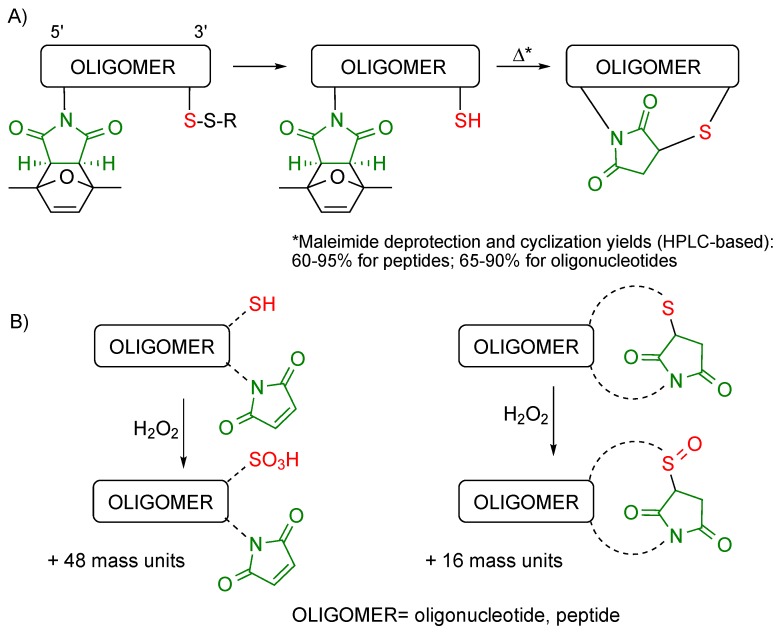
Upon heating, oligonucleotides (and peptides, see below) carrying a protected maleimide and a free thiol undergo an intramolecular Michael-type reaction that furnishes the cyclic compound (**A**). The linear precursor and the cyclic oligomer can easily be distinguished by reaction with hydrogen peroxide and mass spectrometric analysis (**B**).

Our first experiments were carried out with oligonucleotides, which could easily be obtained as linear chains after solid-phase synthesis on CPG enabling thiol derivatization, and incorporation of a protected maleimide at the 5' end, followed by ammonia deprotection (Scheme 14A; oligomer = oligonucleotide) [18].

At first there seemed to be no preferred option for the removal of the thiol and maleimide protecting groups, and both alternatives were tested. Maleimide deprotection as the first step took place satisfactorily. Subsequent removal of the thiol protecting group obviously could not be done by reaction with another thiol, since it would react with the maleimide and prevent cyclization. Therefore, the oligonucleotide was treated with tris-(2-carboxyethyl)phosphine (TCEP). To our surprise, the product resulting from these two reactions, plus incubation at a slightly basic pH to promote cyclization, was neither the linear nor the cyclized oligonucleotide, but an oligonucleotide derivatized with a thiol and a succinimide. It has been described that maleimides can be reduced to succinimides by reaction with triphenylphosphine in methanol [19], and we infer that the same type of reaction is likely responsible for our finding.

The order of the two deprotection reactions was then reversed. TCEP was employed to deprotect the thiol (a thiol could have been used in this case), and subsequently removed, and the resulting oligonucleotide was heated to promote the retro-Diels-Alder reaction to afford a fully deprotected oligonucleotide (Scheme 14A). However, since both the linear precursor and the cyclic molecule have the same mass, we did not know which of these two had been isolated. This question was addressed making use of reaction with H_2_O_2_ (Scheme 14B), which oxidizes the free thiol on the linear molecule to sulfonic acid, and the sulfur of the thiosuccinimide to sulfoxide. These oxidation products can easily be differentiated by mass spectrometric analysis, and the conclusion of these experiments was that after heating the cyclic oligonucleotide had been obtained.

This methodology afforded a variety of cyclic 2'-deoxyoligoribonucleotide sequences, ranging from 5- to 26-mer.

#### 3.2.2. Cyclic and Bicyclic Peptides

For peptide cyclization, peptide-resins incorporating a Trt-protected (Trt = trityl) cysteine residue and either a free maleimide (at the *N* terminus) or a protected maleimide were assembled [20]. Trifluoroacetic acid treatment of the maleimido-(protected peptide)-resin afforded the cyclic peptide, which meant that in the acidic conditions of the final deprotection reaction the linear precursor had cyclized, as assessed by reaction with H_2_O_2_ (see Scheme 14B). Typically, the Michael-type thiol-maleimide reaction is carried at slightly basic pH (7.5–8), to favor thiolate addition to the electrophile while keeping thiol to disulfide oxidation to a minimum. Hence, this result indicates that the reaction can take place even under highly acidic conditions, quite far from conditions commonly accepted as optimal. This synthetic alternative has the disadvantage that impurities (20%–25%) corresponding to cyclic dimer and trimer are obtained in addition to the target cyclic peptide.

The acidic deprotection treatment of (protected maleimido)-(protected peptide)-resins was followed by heating in a MW oven (25–100 μM 1:1 MeOH/H_2_O solutions, 90 min, 90 °C). In these cases, and keeping the concentration of the solutions within the indicated range during maleimide deprotection + cyclization, virtually no undesired dimer and trimer peptides were found (<5%). This approach provided cyclic peptides incorporating a variety of trifunctional amino acids (including histidine) and ranging from 6- to 12-mer.

**Scheme 15:**
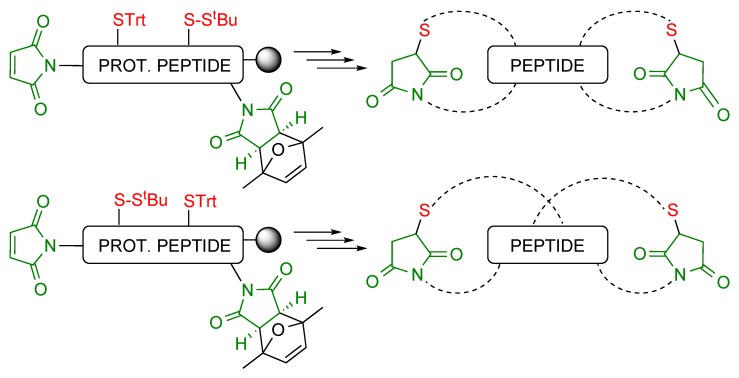
General structures of the bicyclic peptides prepared and the precursor peptide-resins.

**Scheme 16:**
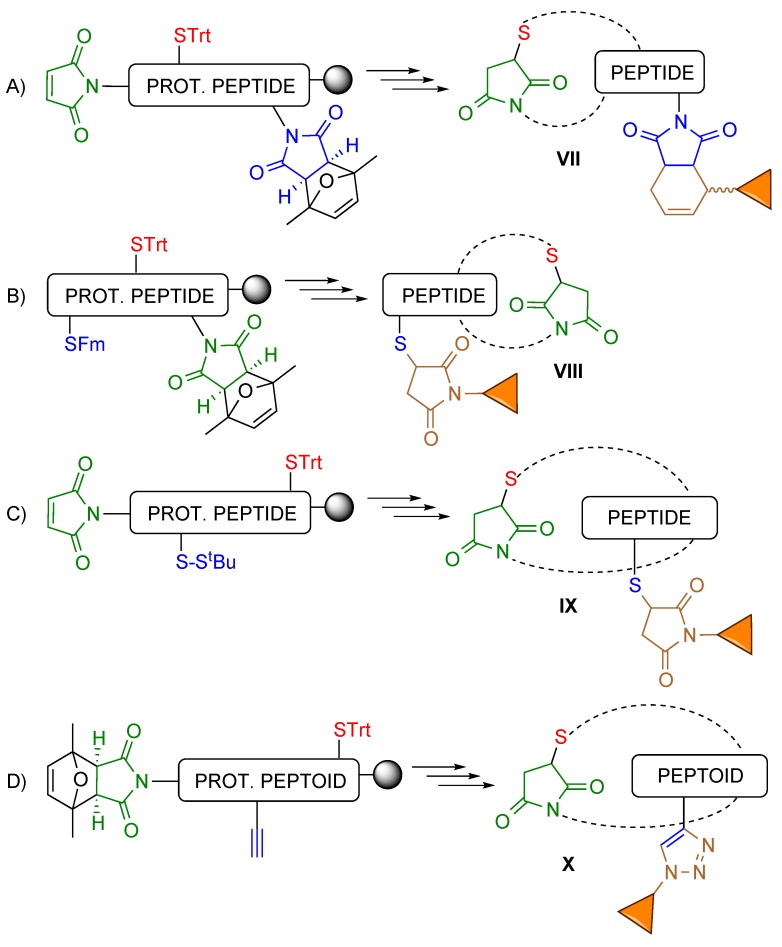
General structure of the precursors of the cyclic peptide/peptoid derivatized for conjugation (the group to be involved in the conjugation reaction is shown in blue), and of the resulting conjugates. The additional moiety can be linked to amino acids not forming the cycle, either at the *C*-terminus (**A**) or at the *N*-terminus (**B**) of the peptide chain, or to internal residues, both in cycles made up of amino acid (**C**) and peptoid (**D**) building blocks.

As described above, maleimides and protected maleimides can be attached to peptides to produce double conjugates. On the other hand, there is a large set of groups to protect the cysteine side chain and be removed under different, orthogonal conditions, which has been exploited to direct the regioselective formation of disulfide bridges [21]. Altogether this opened the possibility of combining maleimides, protected maleimides and differently protected cysteines to prepare a variety of peptides cyclized using the thiol-maleimide Michael-type reaction, such as bicyclic peptides (Scheme 15) and cyclic peptides derivatized for conjugation (Scheme 16, Section 3.2.3). Conjugates of similarly cyclized peptoids have also been obtained.

As shown in Scheme 15, the relative positions of the reacting groups, in particular thiols, determine the structure of the bicyclic peptides, in which either some peptide residues link two isolated cycles, or the two cycles are fused and share amino acids. In any case, chain elongation is followed by the acidic deprotection treatment, which provides the first cycle, removal of the S^t^Bu group with TCEP and purification, and simultaneous maleimide deprotection and cyclization.

#### 3.2.3. Conjugates of Cyclic Peptides and Peptoids

As to the possibility of synthesizing cyclic peptides (or peptoids) incorporating a group that can be used for conjugation to another moiety, Scheme 16 shows what their general structure had to be [22]. The resin-linked peptide (or peptoid) precursor had to incorporate the two groups that would form the cycle plus an additional functionality for conjugation. Again, depending on their relative position the group to be used in the subsequent conjugation reaction would append from either one of the monomers forming the cycle or from an external residue.

The combination of *N*-terminal maleimide, internal Cys(Trt) and protected maleimide at the *C*-terminus of the resin-linked precursor provided a cycle with an appending protected maleimide, and a conjugate with the structure **VII** (Scheme 16A) after simultaneous maleimide deprotection and Diels-Alder conjugation with a 1,3-diene.

A precursor with an *N*-terminal Cys(Fm) (Fm = 9-fluorenylmethyl), an internal Cys(Trt) and a protected maleimide at the *C*-terminus first generated a cycle with an appending protected thiol, from which conjugate **VIII** (Scheme 16B) was obtained after removal of the Fm group with piperidine, reduction of the resulting disulfide with TCEP, and reaction with a maleimide-containing compound.

A cyclic peptide with an internal protected thiol for conjugation was synthesized from a precursor containing an *N*-terminal maleimide, an internal Cys(S^t^Bu) and a *C*-terminal Cys(Trt). In this case the cycle obtained after trifluoroacetic acid-mediated deprotection required treatment with TCEP to deprotect the internal cysteine, and reaction with a maleimide to yield conjugate **IX** (Scheme 16C).

Finally, the cyclic peptoid conjugate **X** (Scheme 16D) was obtained from a (protected peptoid)-resin derivatized at the *N*-terminus with a protected maleimide, and incorporating an alkyne at an internal position and a trityl-protected thiol near the *C*-terminus. Acidic deprotection followed by MW-promoted removal of the maleimide protecting group furnished the cyclic peptoid, and a Cu(I)-catalyzed azide-alkyne Huisgen cycloaddition the desired conjugate.

## 4. Use of Maleimides for Conjugation: Balance and Perspectives

Maleimides are highly reactive electrophiles that quickly react with nucleophiles in Michael-type reactions, and are also very good dienophiles in Diels-Alder cycloadditions. In the absence of any such reagents, they are quite stable in organic solvents, but the range of pH values (5–7) in which they are not quickly degraded is fairly small [23].

The thiol-maleimide click conjugation reaction has many chemical advantages. It does not require catalysts, it is water-compatible, proceeds quickly and cleanly (no side products are generated), and very high to quantitative yields can be achieved with no need for adding large excesses of any of the reagents. Many maleimide-containing compounds are commercially available, but in case they are not, bifunctional compounds incorporating maleimides and carboxyl groups can be easily attached to amines forming amide bonds, either in solution or on a solid phase. Hence, the *N*-terminus of solid-phase assembled polyamides (peptide, PNAs and peptoids to mention only the main ones) can easily be derivatized, and maleimide protection allows maleimide moieties to be introduced at other positions of the polyamide chain as well as to be attached to oligonucleotides. As stated above, maleimido-polyamides are very stable, but in the case of oligonucleotides it is much safer to keep the maleimide protected and deprotect it immediately before use. In any case, it is recommended that best maleimide deprotection conditions are identified in each case. In our hands carrying out maleimide deprotection in the presence of maleimide-reacting compounds such as thiols or 1,3-dienes is the best alternative to reduce the number and amount of byproducts and increase the overall yield.

**Scheme 17:**
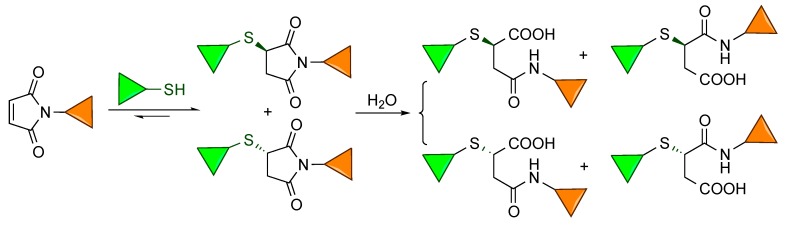
The thiol-maleimide reaction generates thiosuccinimides that may revert to starting materials, as well as undergo hydrolysis. The resulting thiosuccinamic acids are stable.

However, the thiol-maleimide Michael-type reaction generates two diastereomers and not a single product (Scheme 17), and the resulting thiosuccinimide is not fully stable [24]. It can either undergo hydrolysis [25], thiol exchange (through a retro-Michael reaction) [26], or both.

The thiol-maleimide reaction has been commonly employed to prepare antibody-drug conjugates, in particular the two antibody-drug conjugates now approved for the treatment of cancer [2]. In the past few years, and for this particular type of conjugates, evidence has been provided showing that the S-C bond can be cleaved [27], and that the stability of the thiosuccinimide varies depending on the environment [28]. Solvent-accessible linking sites facilitate thiol exchange with plasma thiols, and both accessibility and the charge around the linking site seem to affect imide hydrolysis and/or thiol exchange [29]. Once thiosuccinimides are hydrolyzed and succinamic acid derivatives are generated thiol exchange no longer takes place [26], therefore the use of maleimide derivatives self-promoting hydrolysis of the thiosuccinimide has been proposed as an alternative to stabilize antibody-drug unions [30].

Water-mediated opening of the succinimide ring is an undesired side reaction that leads to a mixture of products (succinimide starting material and two succinamic acids, see Scheme 17), but which does not cleave the covalent linkage between the two components of the conjugate. Succinimide ring opening may also be promoted by non-protonated amines, and has been exploited to attach a new group to a previously formed conjugate [14].

Concerning thiol exchange, the extent of this process (which involves a retro-Michael reaction followed by addition of a new thiol to the transiently formed maleimide) is affected by the pKa of the thiosuccinimide-forming thiol [26]. Since thiosuccinamic acids do not undergo thiol exchange, hydrolysis of the thiosuccinimide may have a beneficial effect on the stability of the conjugate [31].

Cleavage of the carbon-sulfur bond has been observed upon analysis of conjugates by electrophoresis [32,33]. The reported results suggest that thiosuccinimides generated from *N*-arylmaleimides are less stable than those obtained from *N*-alkylmaleimides [32], and are in agreement with the literature [34]. Finally, it has also been described that in thiosuccinimides generated from *N*-alkyl-substituted maleimides the longer the alkyl chain the lower the extent of hydrolysis [30].

Hitherto we have not observed cleavage of the S-C bond, and only on very few occasions hydrolysis of the succinimide ring. After the conjugation reaction, the biotin-PNA conjugate here described was found to be accompanied by some impurities, one of which was the hydrolysis product, and in a previously described experiment we had observed the thiosuccinimide to be highly unstable [16]. Curiously, both conjugates have the presence of the biotin moiety in common, but this is likely a coincidence.

The cycloadducts generated from Diels-Alder conjugation reactions also contain succinimide rings. So far we have not observed hydrolysis of these succinimide rings, and we are not aware of any report disclosing this undesired reaction, which suggests that the stability of succinimides generated from the diene-maleimide reaction is higher than that of thiosuccinimides.

We also wish to emphasize that the two maleimide-involving conjugation reactions we have worked with generate thiosuccinimides and cycloadducts that remain stable under the retro-Diels-Alder conditions that we use to deprotect maleimides. Since the reverse Diels-Alder reaction is carried out at 90 °C, we infer that stability of the two types of succinimides at physiological temperature should not be a concern.

To sum up, the Diels-Alder cycloaddition furnishes fairly stable conjugates, but different cycloadducts may also be formed. Reaction times are longer than those needed for the thiol-maleimide reaction, and heating may eventually be required. The Michael-type thiol-maleimide reaction is not devoid of problems, but it remains a powerful alternative to be taken into consideration, especially in experiments not requiring the linkage between the conjugate components to be permanently stable. The reaction is quick and efficient, the reagents are easy to use, and many thiol and maleimide-containing derivatives are commercially available. In case they are not, many possibilities have been described to easily attach maleimides or protected maleimides to the desired molecule. As reviewed here, maleimide protection contributes to broaden the scope of alternatives for the modification of biomolecules and analogs.

Finally, a discussion of the advantages and disadvantages of maleimide-involving conjugation reactions in which thiols are important players could not be concluded without mentioning that different groups are working in the development of thiol-involving reactions that furnish stable conjugates [35,36].
